# Monocular 3D Multi-Person Pose Estimation for On-Site Joint Flexion Assessment: A Case of Extreme Knee Flexion Detection

**DOI:** 10.3390/s24196187

**Published:** 2024-09-24

**Authors:** Guihai Yan, Haofeng Yan, Zhidong Yao, Zhongliang Lin, Gang Wang, Changyong Liu, Xincong Yang

**Affiliations:** 1 Central Research Institute of Building and Construction Co., Ltd., MCC Group, Shenzhen 518088, China; yanguihai@cribc.com (G.Y.); yaozhidong@cribc.com (Z.Y.); linzhongliang@cribc.com (Z.L.); 2 School of Civil Engineering, Harbin Institute of Technology, Harbin 150090, China; liuchangyong@hit.edu.cn; 3 China Jingye Engineering Technology, Co., Ltd., Shenzhen 518055, China; wanggang@szsti.org; 4 School of Civil and Environmental Engineering, Harbin Institute of Technology, Shenzhen 518055, China; yangxincong@hit.edu.cn; 5 Guangdong Provincial Key Laboratory of Intelligent and Resilient Structures for Civil Engineering, Shenzhen 518055, China

**Keywords:** 3D pose estimation, multi-person, ergonomic risk assessment, construction safety, computer vision, deep learning

## Abstract

Work-related musculoskeletal disorders (WMSDs) represent a significant health challenge for workers in construction environments, often arising from prolonged exposure to ergonomic risks associated with manual labor, awkward postures, and repetitive motions. These conditions not only lead to diminished worker productivity but also incur substantial economic costs for employers and healthcare systems alike. Thus, there is an urgent need for effective tools to assess and mitigate these ergonomic risks. This study proposes a novel monocular 3D multi-person pose estimation method designed to enhance ergonomic risk assessments in construction environments. Leveraging advanced computer vision and deep learning techniques, this approach accurately captures and analyzes the spatial dynamics of workers’ postures, with a focus on detecting extreme knee flexion, a critical indicator of work-related musculoskeletal disorders (WMSDs). A pilot study conducted on an actual construction site demonstrated the method’s feasibility and effectiveness, achieving an accurate detection rate for extreme flexion incidents that closely aligned with supervisory observations and worker self-reports. The proposed monocular approach enables universal applicability and enhances ergonomic analysis through 3D pose estimation and group pose recognition for timely interventions. Future efforts will focus on improving robustness and integration with health monitoring to reduce WMSDs and promote worker health.

## 1. Introduction

Work-related musculoskeletal disorders (WMSDs) are among the most common occupational illnesses in the construction industry and constitute a significant cause of non-fatal injuries to workers [1]. WMSDs not only inflict physical pain and suffering on workers but also result in absenteeism, project delays, and increased financial burdens on construction projects [2,3]. Various factors contribute to the development of WMSDs, including awkward postures, repetitive tasks, prolonged working hours, and individual characteristics such as age and previous WMSD history. Among these, awkward postures, such as bending, squatting, and kneeling, can pose serious ergonomic risks [4]. Correcting these awkward postures is an effective intervention strategy to reduce the risk of WMSDs [5]. However, in practice, without an automated real-time posture assessment system, it is challenging for supervisors to remind workers to take breaks or adjust their working postures. Therefore, accurate and efficient ergonomic posture assessment is a crucial prerequisite for proactive ergonomic risk management.

Significant research has been conducted in the field of ergonomic risk assessment to promote occupational health and safety. Traditional methods often involve manual observations and subjective assessments, which can be time-consuming and labor-intensive [6]. Recent advancements in computer vision technologies offer promising solutions for ergonomic assessments. Visual-based systems, particularly those utilizing pose estimation algorithms, provide objective, accurate, and real-time monitoring capabilities. These systems enable continuous assessment of workers’ postures without interrupting their activities, thus enhancing the effectiveness of ergonomic interventions.

However, traditional monocular two-dimensional pose detection methods face limitations in accuracy due to the inconsistency of detected joint angles from different viewpoints, which is crucial for ergonomics research. While three-dimensional pose detection systems (such as those using sensors or reflective markers for body motion capture) can provide more accurate spatial information, they may face challenges such as the need for depth sensors or multi-ocular camera systems to derive depth information through stereo matching. Depth sensors are costly, and stereo matching demands significant computational resources. Additionally, installing such equipment at construction sites may not be feasible for all projects [7].

Therefore, to address these challenges, we propose a monocular 3D multi-person pose estimation method specifically designed for construction sites. Monocular cameras, which are often already installed for security purposes, offer a convenient and cost-effective solution for capturing high-resolution images. This approach is well suited to the dynamic and crowded nature of construction sites, allowing for the simultaneous monitoring of multiple workers. What is more, compared to 2D pose estimation, 3D pose estimation offers a more comprehensive analysis of workers’ postures, facilitating better safety monitoring and risk assessment. By integrating these advanced technologies into existing site infrastructure, our method enables automated ergonomic evaluations, significantly improving the health and safety of construction workers.

## 2. Related Works

### 2.1. Ergonomic Risk Assessment in Construction

In the construction industry, it is widely known that work-related musculoskeletal disorders (WMSDs) are alarmingly prevalent among workers, which has long been a matter of great concern. Despite the fact that numerous studies have been carried out with the aim of thoroughly understanding these risks and finding ways to mitigate them, substantial challenges still persist. For instance, Mohammadiyan et al. delved into the prevalence of WMSDs and successfully identified crucial risk factors among construction workers [8]. Rabbani et al. emphasized the significance of human factors in reducing WMSDs and strongly advocated for the application of ergonomic analysis tools [9]. Carpio et al. meticulously developed a protocol to classify preventive action levels in construction works [10], while Wang et al. put forward a 3D fuzzy ergonomic analysis method for the purpose of rapid workplace design and modification to address ergonomic risks [11]. Seo et al. [12] introduced an innovative vision-based method for automated postural ergonomic risk assessment, and Rodrigues et al. [13] conceptualized a self-assessment tool named microErgo to reduce ergonomic risks. Palikhe et al. [14] conducted a detailed analysis of WMSDs in aluminum form workers at construction workstations, and Vijayakumar et al. [15] carried out a scientometric visualization analysis on the emerging trends in ergonomic risk assessment in construction safety management. Tao et al. [16] proposed an ergonomic risk assessment method based on a fuzzy Bayesian network and D-S evidence theory [17] specifically for construction workers and projects.

However, despite these valuable contributions, the field still encounters several limitations. One of the major issues is that the integration of ergonomic solutions into actual construction practices remains inconsistent and far from ideal. The complex and dynamic nature of construction tasks often undermines the effectiveness of current interventions. Moreover, many existing methods frequently suffer from a lack of comprehensive validation and practical applicability. As a result, their effectiveness in reducing work-related musculoskeletal disorders (WMSDs) is significantly limited, which poses a significant obstacle to the improvement of workers’ health and safety in the construction industry.

### 2.2. Human Pose Estimation

Human pose estimation is an essential and crucial task within the field of computer vision, possessing a wide array of applications that span from human–computer interaction to surveillance and sports analytics. The recent remarkable advancements in deep learning have substantially enhanced the accuracy and efficiency of pose estimation methods [18]. Since 2014, deep learning-based approaches have made extraordinary progress in both 2D and 3D human pose estimation, effectively handling challenges such as scale variation, depth ambiguities, and occlusions [19,20].

One of the commonly used methods in human pose estimation is the bottom-up approach. Despite its proven effectiveness, it encounters difficulties in predicting poses for smaller individuals due to the problem of scale variation [19]. To address this issue, HigherHRNet was introduced as a novel bottom-up method that emphasizes learning scale-aware representations via high-resolution feature pyramids [19]. On the contrary, Moon et al. proposed a camera distance-aware top-down approach for 3D multi-person pose estimation from a single RGB image. This fully learning-based method enhances accuracy by taking into account the distance of the camera [21].

Chen et al. concentrated on monocular human pose estimation and emphasized the substantial progress achieved through deep learning techniques [18]. In the domain of multi-camera 3D human pose estimation, Tu et al. introduced VoxelPose [22], an end-to-end solution that operates directly in 3D space, eliminating the necessity for cross-view correspondence based on 2D pose estimations. Additionally, Artacho et al. proposed UniPose [23], a unified framework that achieved state-of-the-art results in both single images and videos. Recent innovative efforts, such as PoseFormer, have explored transformer-based approaches for 3D human pose estimation in videos [24]. By focusing on the spatial and temporal joint relations, these methods are able to produce more accurate 3D poses.

Despite these advancements, several key challenges remain unresolved. Firstly, monocular pose estimation still encounters significant challenges because of depth ambiguities and occlusions. Current methods frequently have difficulties in accurately inferring the 3D position of joints from a single camera view. Secondly, although multiple camera systems can provide more accurate 3D pose estimations, they are often impractical for real-world applications due to their complexity and high cost. Finally, group pose recognition, especially in dynamic and crowded environments, remains a difficult task. The presence of overlapping and interacting individuals complicates the estimation process, making it challenging to accurately estimate the poses of each individual within the group.

## 3. Methodology

Effective prevention of work-related musculoskeletal disorders (WMSDs) is critical in ensuring the health, safety, and productivity of construction workers. These disorders, often caused by repetitive strain, awkward postures, and overexertion, can lead to chronic pain and long-term disability. Monitoring and analyzing workers’ 3D postures in real time provides valuable insights into the risk factors associated with WMSDs, enabling the implementation of proactive measures to mitigate these risks. Accurate detection and evaluation of joint angles are essential in identifying potentially harmful postures, thereby preventing injuries and promoting safer working conditions.

To address these challenges, we have developed a robust and efficient method for posture assessment and awkward posture detection among construction workers using monocular 3D multi-person pose estimation.

### 3.1. Workflow of the Proposed Method

**Figure 1:**
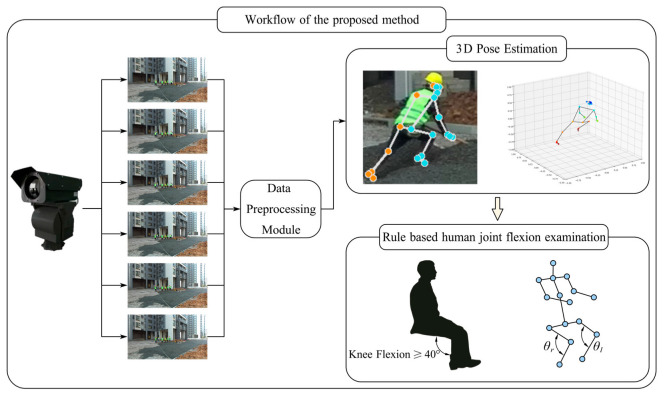
Workflow of the proposed method.

In this study, we propose a comprehensive method for monocular 3D multi-person pose estimation and awkward posture detection for construction workers, leveraging image data captured by a single monocular camera. The workflow of the proposed method consists of several key components, as shown in Figure 1, each designed to enhance the accuracy and reliability of posture assessment in a dynamic construction site environment.

Image Capture: High-resolution images are collected using strategically positioned monocular cameras across the construction site. These cameras operate continuously during work hours, capturing images at a fixed frame rate.

Data Preprocessing Module: The raw image data undergo a series of preprocessing steps to improve the visibility of key features and reduce noise. This includes histogram equalization for contrast adjustment, adaptive thresholding to manage varying light intensities, Gaussian blurring for noise reduction, and normalization to ensure consistent input dimensions.

Monocular 3D Pose Estimation: Preprocessed images are fed into a monocular 3D pose estimation model. This model detects the 3D coordinates of key body joints and skeletal structures, providing a comprehensive representation of the worker’s posture.

Awkward Posture Detection: Based on the identified 3D key points and skeletal information, the system calculates the corresponding joint angles. These angles are then evaluated against the criteria set forth by the International Organization for Standardization (ISO) to detect awkward postures. Specific thresholds are defined for various joints to determine whether a posture deviates significantly from the ergonomic standards.

In summary, the proposed method integrates state-of-the-art techniques in image processing, deep learning, and ergonomic analysis to provide a reliable and efficient solution for 3D human pose estimation and awkward posture detection in construction environments. This approach not only improves the accuracy of posture assessments but also contributes to the proactive management of worker health and safety.

### 3.2. Data Acquisition and Preprocessing

#### 3.2.1. Data Acquisition

In this study, we employ a monocular camera setup to capture image data for the purpose of monocular 3D multi-person pose estimation among construction workers. The camera is strategically positioned at various locations within the construction site to ensure comprehensive coverage of the workers’ activities. The selection of the camera positions is based on a preliminary site analysis, considering factors such as worker density, activity types, and potential occlusions.

The surveillance camera system operates continuously during working hours, capturing high-resolution images at a frame rate of 30 frames per second (fps). To ensure data reliability and consistency, we synchronize the camera system with the site’s operational schedule and environmental conditions. All captured images are timestamped and geotagged to facilitate subsequent data alignment and analysis.

#### 3.2.2. Data Preprocessing

**Figure 2:**
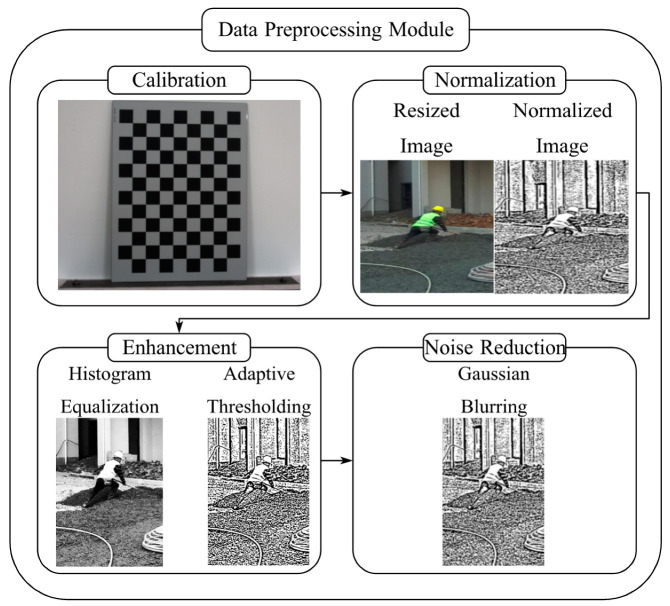
Data Preprocessing Module.

The raw image data obtained from the monocular camera undergo an array of preprocessing procedures for the 3D pose estimation task, as depicted in Figure 2. Taking these steps is of paramount importance to guarantee the quality and precision of the pose estimation process.

1. Image Calibration:

Before deployment, the monocular camera is calibrated with a standard checkerboard pattern to ascertain the intrinsic and extrinsic parameters [25]. Conducting this calibration process is indispensable for rectifying lens distortions and ensuring accurate 3D reconstruction from 2D images.

2. Input Data Normalization:

The images are resized to a unified resolution to ensure consistent input dimensions for the pose estimation model. Additionally, pixel values are normalized to a [0, 1] range to standardize the input data. This normalization step not only simplifies the subsequent processing but also helps to improve the convergence and stability of the model.

3. Image Enhancement:

In light of the variable lighting conditions on the construction site, image enhancement techniques are employed to enhance the visibility of key features. These techniques encompass histogram equalization to adjust the contrast and adaptive thresholding to handle varying light intensities.

4. Noise Reduction:

Construction sites are frequently prone to dust and other particulate matter, which can introduce noise into the images. To alleviate this issue, Gaussian blurring is applied to reduce high-frequency noise while preserving the essential structural information of the workers’ poses.

The preprocessed high-quality and normalized images are ready for the subsequent 3D pose estimation task. The preprocessing pipeline ensures that the model receives clean and consistent input, thereby enhancing its performance and reliability in real-world construction site conditions.

### 3.3. Computer Vision-Based Monocular Multi-Person 3D Pose Estimation

In this study, we employed a state-of-the-art monocular 3D multi-person pose estimation algorithm to accurately detect and analyze the postures of construction workers. The algorithm is based on the lightweight OpenPose [26] and the single-shot method for multi-person 3D pose estimation [27].

#### 3.3.1. Algorithm Overview

Our pose estimation method initially leverages the lightweight OpenPose architecture to detect the 2D coordinates of up to 18 key points on the human body, including the ears, eyes, nose, neck, shoulders, elbows, wrists, hips, knees, and ankles. This 2D detection process is optimized for real-time performance on CPU platforms, making it highly suitable for deployment in dynamic and resource-constrained construction site environments.

Subsequently, the algorithm utilizes a single-shot approach to estimate the 3D coordinates of these key points. The single-shot method for multi-person 3D pose estimation is aimed at analyzing various scenes with a monocular RGB camera. This advanced technique incorporates occlusion-robust pose maps (ORPMs), facilitating precise full-body pose detection, even under considerable partial occlusions caused by other people or objects. ORPMs produce a predetermined set of maps that capture the 3D joint positions for all individuals in the scene. By leveraging body part associations, this approach can determine the 3D poses of multiple individuals without requiring specific bounding box predictions. This approach not only simplifies the system setup but also enhances its practicality and ease of deployment in real-world scenarios.

#### 3.3.2. Inference and Performance

The pose estimation model uses the OpenVINO pre-trained model weights, which are rigorously trained on the MS COCO and CMU Panoptic datasets. The MS COCO dataset provides extensive annotations for 2D key points across a wide array of human activities, while the CMU Panoptic dataset offers detailed 3D joint positions captured in controlled environments. The combination of these datasets ensures that the model is robust and capable of generalizing well to various real-world scenarios, including the diverse and unpredictable conditions of construction sites.

The model achieves a mean per joint position error (MPJPE) of 100 mm on the CMU Panoptic subset. This metric, representing the average deviation of the predicted joint positions from the ground truth, underscores the model’s precision in 3D human pose estimation.

### 3.4. Human Joint Flexion Examination

The exact angles of human joints are closely linked to work-related musculoskeletal disorders (WMSDs). For instance, extreme flexion of the knee joint imposes excessive stress on the knees, potentially leading to knee disorders. The knee joint’s extreme flexion, which is common in various construction tasks, is ergonomically undesirable according to ISO standards (ISO 11226:2000) [28]. These standards define thresholds for joint angles that, if exceeded, could increase the risk of injury. By studying the variations in these key joint angles, we can better understand and prevent the occurrence of WMSDs. This, in turn, enables the optimization of working postures and movements, reducing the burden on the musculoskeletal system and enhancing both worker health and productivity. Therefore, in-depth research into the precise angles of key joints is crucial for developing effective preventive strategies and improving workplace conditions.

The extraction of skeletal information allows for the straightforward computation of corresponding joint angles. These calculations rely on the coordinates of skeletal key points obtained from pose estimation models. For instance, the angle of any joint can be determined using specific geometric formulas based on the positions of the surrounding key points. Below is a general formula for calculating the joint angles.

Assume the coordinates of the key points forming a joint are represented as A, B, and C, where B is the joint in question and A and C are the adjacent points. The coordinates are given by the following:

(1) $$A=(x_{1},y_{1})$$

(2) $$B=(x_{2},y_{2})$$

(3) $$C=(x_{3},y_{3})$$

To compute the angle θ at the joint B, we use the vectors formed by these points:

(4) $$\vec{u}=A\;-\;B=(x_{1}-x_{2},y_{1}-y_{2})$$

(5) $$\vec{v}=C\;-\;B=(x_{3}-x_{2},y_{3}-y_{2})$$

The angle between these vectors can be calculated using the following formula:

(6) $$\cos(\theta )=\frac{\vec{u}\cdot \vec{v}}{\vec{\left|u\right|}\vec{\left|v\right|}}$$

where the dot product $\vec{u}\cdot \vec{v}$ and the magnitudes $\vec{\left|u\right|}$ and $\vec{\left|v\right|}$ are computed as

(7) $$\vec{u}\cdot \vec{v}=(x_{1}-x_{2})(x_{3}-x_{2})+(y_{1}-y_{2})(y_{3}-y_{2})$$

(8) $$\vec{\left|u\right|}=\sqrt{{\left(x_{1}-x_{2}\right)}^{2}+{\left(y_{1}-y_{2}\right)}^{2}}$$

(9) $$\vec{\left|v\right|}=\sqrt{{\left(x_{3}-x_{2}\right)}^{2}+{\left(y_{3}-y_{2}\right)}^{2}}$$

Finally, the joint angle θ can be determined by taking the arccosine of the cosine value:

(10) $$\theta =\arccos(\frac{\vec{u}\cdot \vec{v}}{\vec{\left|u\right|}\vec{\left|v\right|}})$$

This general formula can be applied to calculate the angle of any joint by substituting the appropriate key point coordinates.

If $\theta <\theta_{\mathrm{threshold}}$, where $\theta_{\mathrm{threshold}}$ is the critical angle defined by ergonomic standards, then extreme joint flexion will be detected.

## 4. Pilot Study

To illustrate the process and demonstrate its applicability, we conducted a pilot study using video data collected from an actual construction project. A monocular camera was strategically installed on-site, capturing real-time footage of workers at 30 frames per second. Over the course of the study, we accumulated a total of 30,000 frames of on-site data. We used the S-YUE X5 (1080P) monocular camera for the pilot study (Shenzhen Vxinstae Technology Co., Shenzhen, China). Our vision-based method works effectively within a camera field of view of up to 20 m, achieving optimal recognition and monitoring within this range.

The collected data underwent several preprocessing steps using our Data Preprocessing Module to enhance quality and reduce noise. These preprocessed data were then fed into our Computer Vision-Based Multi-Person 3D Pose Estimation model to perform group 3D pose recognition. The model was deployed on a 64-bit Windows system and implemented using Python 3.8 and the PyTorch platform. The hardware configuration included an Intel(R) Core (TM) i7-14700K @ 3.40 GHz processor, 128 GB of RAM, and an NVIDIA GeForce RTX 4080 SUPER @ 16 GB GPU (Harbin Institute of Technology, Shenzhen, China; Intel, Shenzhen, China; Kingston Technology; Fountain Valley, CA, USA; Asus, Taipei, Taiwan).

**Figure 3:**
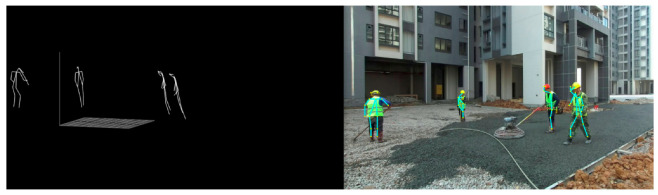
Results of the Computer Vision-Based Multi-Person 3D Pose Estimation.

Figure 3 shows the output of our 3D pose estimation, highlighting the accurate detection of various body joints and their movements. In the figure, the right side displays the actual image of the construction site, while the left side shows the recognized 3D key points of the worker group.

**Figure 4:**
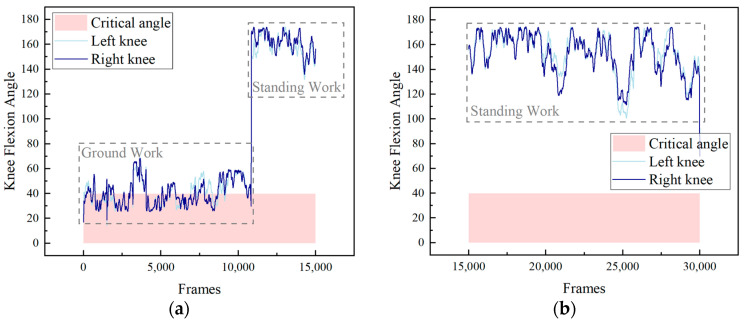
Knee flexion angle of the worker. (**a**) Knee angle of 0–15,000 frames. (**b**) Knee angle of 15,000–30,000 frames.

Using the knee angle during construction as a case study, we employed the Computer Vision-Based Multi-Person 3D Pose Estimation method and Formula (10) to calculate the knee joint angles throughout the construction process. These angles are crucial for assessing ergonomic risks, particularly in detecting extreme knee flexion. Figure 4 illustrates the knee joint angles of one worker’s left and right knees on the construction site, with the light red area representing the critical angle threshold defined by ergonomic standards. In the diagram, workers’ activities are distinguished and labeled with dotted boxes to indicate the type of work they are performing.

During the observed construction activity, a total of four workers were analyzed. Detailed experimental results are presented in Table 1. The second column of the table indicates the maximum knee angle of each worker, and the third column presents the minimum knee angle of each worker. The last column is the duration of extreme flexion of each worker during the pilot study.

The average maximum knee angle of the four workers is 174.3°, while the average minimum knee angle is 16.7°. Among the four workers, Worker 1 had the smallest minimum knee angle, indicating a more severe degree of extreme flexion. Prolonged periods of extreme knee flexion can further exacerbate the risk of WMSDs. Worker 4′s extreme flexion lasted the longest and required attention, whereas Worker 3′s duration was the shortest. These findings closely corresponded with the observations made by on-site supervisors and the self-reported discomfort expressed by the workers.

These findings provide valuable insights into the lower body ergonomics of construction workers. By identifying and quantifying the instances of awkward postures, such as extreme knee flexion, our study offers empirical evidence that can be used to design effective posture intervention and correction strategies. This approach not only helps in mitigating the risk of WMSDs but also contributes to the overall improvement of worker health and safety on construction sites. For example, the identification of Worker 1′s extreme knee flexion suggests the need for targeted intervention to prevent potential WMSDs, which could include ergonomic training or the redesign of tasks and tools. Similarly, the prolonged extreme flexion observed in Worker 4 underscores the necessity for regular breaks or adjustments in work schedules to reduce the risk of injury. Additionally, the shorter duration of extreme flexion in Worker 3 raises questions about possible differences in individual work habits or task assignments, which could inform more personalized ergonomic solutions.

In conclusion, our pilot study demonstrates the feasibility and effectiveness of using monocular 3D multi-person pose estimation for ergonomic assessment in construction environments. The ability to capture and analyze detailed joint movements provides a robust foundation for proactive ergonomic risk management, ultimately enhancing workplace safety and productivity.

## 5. Conclusions and Future Work

The proposed monocular 3D multi-person pose estimation method has been successfully developed and implemented to address ergonomic risks among construction workers. By employing advanced computer vision and deep learning techniques, our method has effectively captured and analyzed the 3D postures of workers, focusing particularly on the detection of extreme flexion in knee joints, a critical risk factor for work-related musculoskeletal disorders (WMSDs).

The pilot study conducted on an actual construction site has demonstrated the feasibility and effectiveness of our approach. The method, which can be integrated seamlessly into the existing site infrastructure, has yielded promising results in ergonomic assessment. Specifically, it has accurately evaluated the joint angles of the four workers during construction activities. This aligns closely with supervisory observations and worker self-reports, validating the method’s capability to provide actionable insights for proactive risk management.

Despite the encouraging outcomes, this pilot study has highlighted areas for improvement and further research:

(1) Handling Occlusions and Overlapping: The current method struggles with occlusions and overlapping among workers, which can lead to inaccurate pose estimations. For example, if workers are in a crowded work area with many objects or other workers blocking the view of certain body parts, the accuracy of pose estimation can be reduced. Future enhancements will explore multi-view fusion techniques to mitigate these issues, potentially integrating data from multiple cameras to enhance the robustness and accuracy of pose detection.

(2) Scalability and Integration: The scalability of the method to larger construction sites and its compatibility with other health and safety monitoring systems will be a focus of future research. This includes developing strategies for efficient deployment across diverse construction environments and integrating additional health monitoring features to provide comprehensive worker health assessments.

In conclusion, the monocular 3D multi-person pose estimation method represents a significant advancement in ergonomic risk management in construction. Its universal applicability, enhanced 3D analysis, and group detection capabilities position it as a powerful tool for improving worker safety and health. Continued research and development will ensure that the system remains at the forefront of ergonomic technology, contributing to a safer and more productive construction industry.

## Figures and Tables

**Table 1:** Results of the pilot study.

Worker	Maximum Knee Angle (Degrees)	Minimum Knee Angle (Degrees)	Duration of Extreme Flexion (s)
1	174.5°	14.2°	191
2	174.4°	17.0°	205
3	174.0°	18.3°	178
4	174.3°	17.1°	219
Average	174.3°	16.7°	198.25

## Data Availability

Some or all data, models, or codes that support the findings of this study are available from the corresponding author upon reasonable request.
